# Nutritive Value of 11 Bee Pollen Samples from Major Floral Sources in Taiwan

**DOI:** 10.3390/foods10092229

**Published:** 2021-09-20

**Authors:** Pei-Shou Hsu, Tzu-Hsien Wu, Meng-Yuan Huang, Dun-Yan Wang, Ming-Cheng Wu

**Affiliations:** 1 Department of Entomology, College of Agriculture and Natural Resources, National Chung Hsing University, Taichung 402202, Taiwan; pshsu@mdais.gov.tw (P.-S.H.); thwu@mdais.gov.tw (T.-H.W.); rocker840308@gmail.com (D.-Y.W.); 2 Miaoli District Agricultural Research and Extension Station, Council of Agriculture, Executive Yuan, Miaoli 363201, Taiwan; 3 College of Life Sciences, National Chung Hsing University, Taichung 402202, Taiwan; hmy6@nchu.edu.tw

**Keywords:** bee pollen, macronutrients, amino acid, fatty acid

## Abstract

Bee pollen is a nutrient-rich food that meets the nutritional requirements of honey bees and supports human health. This study aimed to provide nutritive composition data for 11 popular bee pollen samples (*Brassica napus* (Bn), *Bidens pilosa* var. *radiata* (Bp), *Camellia sinensis* (Cs), *Fraxinus griffithii* (Fg), *Prunus mume* (Pm), *Rhus chinensis* var. *roxburghii* (Rc), *Bombax ceiba* (Bc), *Hylocereus costaricensis* (Hc), *Liquidambar formosana* (Lf), *Nelumbo nucifera* (Nn), and *Zea mays* (Zm)) in Taiwan for the global bee pollen database. Macronutrients, such as carbohydrates, proteins, and lipids, were analyzed, which revealed that Bp had the highest carbohydrate content of 78.8 g/100 g dry mass, Bc had the highest protein content of 32.2 g/100 g dry mass, and Hc had the highest lipid content of 8.8 g/100 g dry mass. Only the bee pollen Hc completely met the minimum requirements of essential amino acids for bees and humans, and the other bee pollen samples contained at least 1–3 different limiting essential amino acids, i.e., methionine, tryptophan, histidine, valine, and isoleucine. Regarding the fatty acid profile of bee pollen samples, palmitic acid (C_16:0_), stearic acid (C_18:0_), oleic acid (C_18:1_), linoleic acid (C_18:2_), and linolenic acid (C_18:3_) were predominant fatty acids that accounted for 66.0–97.4% of total fatty acids. These data serve as an indicator of the nutritional quality and value of the 11 bee pollen samples.

## 1. Introduction

Bee pollen is a valuable natural product containing at least 200 biologically active substances with potential therapeutic applications [1,2,3]. It is produced by beekeepers and used as the primary nutrition source for a honey bee colony’s proper growth and development. Additionally, the comprehensive nutrition of bee pollen can meet the requirements of humans. With a rapidly growing older population, the demand for healthy food supplements is increasing. Therefore, bee pollen is a highly consumed natural product as a dietary supplement for humans [3]. Moreover, bee pollen is recommended as a food supplement for livestock because its composition is beneficial for their health. For example, bee pollen can enhance the growth performance, reproduction, and immunity of animals [4]. Accordingly, bee pollen has gained increasing research attention worldwide [3,5,6,7,8,9,10,11]. Pollen composition considerably varies depending on its floral and geographic origins. In terms of macronutrients, pollen contains approximately 2–60% of proteins, 1–20% of lipids, and 13–55% of carbohydrates [3,12,13,14,15,16]. The quality and diversity of pollen diets have been closely linked to bee and human health [17,18,19].

The protein content of bee pollen can be used as an index of pollen nutritional value [12,14,15]. However, bee pollen obtained from different floral sources differ in their amino acid composition [3,14,16,20,21]. Inadequate amounts of essential amino acids (EAAs) can reduce the nutritional value of bee pollen [5,18,22,23]. Therefore, its nutritional value must be determined by investigating its amino acid composition [23]. In an early study, De Groot [24] showed that 10 amino acids, namely arginine, histidine, isoleucine, leucine, lysine, methionine, phenylalanine, threonine, tryptophan, and valine, were EAAs for honey bees with minimum requirements of 3.0, 1.5, 4.0, 4.5, 3.0, 1.5, 1.5, 1.5, 1.0, and 4.0 g/100 g proteins, respectively. In addition, the minimum requirements of amino acids for humans, namely histidine, isoleucine, leucine, lysine, methionine, threonine, tryptophan, valine, and phenylalanine–tyrosine, are 1.5, 3.0, 5.9, 4.5, 1.6, 2.3, 0.6, 3.9, and 3.8 g/100 g proteins [25].

Bee pollen is an essential source of fatty acids for honey bees. It not only is used for energy and as a structural component of cell membranes, but also plays crucial roles in bee health and behavior [26,27,28,29,30,31,32]. Generally, the fatty acid content of pollen is dominated by saturated fatty acids, such as myristic acid (C_14:0_), palmitic acid (C_16:0_), and stearic acid (C_18:0_), as well as unsaturated fatty acids, such as oleic acid (C_18:1_), linoleic acid (C_18:2_), and alpha-linolenic acid (C_18:3_) [26]. Among fatty acids, polyunsaturated fatty acids (PUFAs) cannot be synthesized by honey bees and humans. Therefore, two PUFAs, linoleic acid (omega-6 fatty acid) and alpha-linolenic acid (omega-3 fatty acid), are often considered to be essential fatty acids [32].

Taiwan has a blooming beekeeping industry because of its unique geographic location and its tropical and subtropical climate. Thus, Taiwan has abundant and diverse nectariferous plants [33,34]. Currently, approximately 160,000 managed colonies of *Apis mellifera* in Taiwan can produce at least 700 tons of bee pollen per year according to the records of the Taiwan beekeeping association. The major floral sources of pollen for honey bees in Taiwan are rape (*Brassica napus*, Bn), beggartick (*Bidens pilosa* var. *radiata*, Bp), tea tree (*Camellia sinensis*, Cs), evergreen ash (*Fraxinus griffithii*, Fg), Japanese apricot (*Prunus mume*, Pm), nutgall tree (*Rhus chinensis* var. *roxburghii*, Rc), red cotton tree (*Bombax ceiba*, Bc), pitaya (*Hylocereus costaricensis*, Hc), Formosan gum (*Liquidambar formosana*, Lf), lotus (*Nelumbo nucifera*, Nn), and corn (*Zea mays*, Zm). Bee pollen obtained from these sources accounts for most of the total annual pollen collected for commercial usage. However, their nutritional value has not yet been studied. This research mainly focused on investigating the macronutrient, amino acid, and fatty acid contents of 11 monofloral bee pollen samples and presented their nutritional values in terms of the requirements of honey bees and humans.

## 2. Materials and Methods

### 2.1. Bee Pollen Sample Collection

We cooperated with Yong Shyang Apiculture Co., Ltd. (Chia Yi City, Taiwan) to collect 11 floral bee pollen samples from Taiwanese local beekeepers in 2019 and 2020. Generally, pollen traps were used to collect bee pollen in the morning of days with favorable weather conditions and were emptied at around 4 pm. The harvested bee pollen was immediately frozen (below 0 °C) and stored until transportation to the laboratory. In the laboratory, bee pollen was manually refined on the basis of color, and the pollen of the same color was pooled. These pooled samples were checked for their purity and botanical origin through light microscopy (Axio Scope A1, Zeiss, China). Purity was analyzed by calculating the percentage of the main species of pollen grains present in 1 g of each sample [16]. The purities of all samples were found to be >98%. Plant species were first identified based on palynological keys [35], with further examination of sequenced *rbcL* and *trnH-psbA* marker genes. The gene *rbcL* was amplified using primers *rbcLa*F (A T G T C A C C A C A A A C A G A G A C T A A A G C) and *rbcL*ar590 (A T G A A T G T C T A C G C G G T G G A C T) [36,37]. The *trnH-psbA* region was amplified using primers *psbA* (G T T A T G C A T G A A C G T A A T G C T C) and *trnH* (C G C G C A T G G T G G A T T C A C A A T C C) [38,39]. Finally, the samples were sieved through a stainless steel 35 wire mesh (0.5-mm), manually pulverized for 1 min, repacked, and frozen at −18 °C for further chemical analysis. In this study, 23 bee pollen samples from 11 floral origins were collected for analysis. Each of six kinds of floral bee pollen samples, i.e., Bn, Bp, Cs, Fg, Pm, and Rc, were collected from three different places, respectively. The other five bee pollen samples, namely Bc, Hc, Lf, Nn, and Zm, were only collected from one place due to the lack of availability of botanical origins.

### 2.2. Proximate Analysis of Bee Pollen

The proximate compositions of water, protein, lipid, and ash were analyzed according to the National Standards of the Republic of China (CNS) authorized by the Bureau of Standards, Metrology and Inspection, Ministry of Economic Affairs, R.O.C. The fresh bee pollen samples (about 200 g) were drying in an oven at 40 °C for at least 24 h until a constant weight was obtained. The water content was calculated by comparing the weight of fresh and dehydrated bee pollen samples [40]. However, 4 of 23 bee pollen samples (2 of Fg, 1 of Pm, and 1 of Rc) were pre-dried by beekeepers. Accordingly, the water content of these four samples was not included in the results. Subsequently, the protein, lipid, and ash contents of the dried bee pollen were analyzed. The protein content was determined using the Kjedahl method, and the total protein content was obtained by multiplying nitrogen values by a conversion factor of 6.25 [41]. The lipid content was determined using a Soxhlet extractor with diethyl ether as the solvent [42]. The ash content was measured using the dry combustion method in a 600 °C furnace [43]. The results are reported as grams per 100 g of dry bee pollen, except for water content, which is reported as grams per 100 g of fresh bee pollen. The carbohydrate content was calculated as follows: carbohydrate content = 100 − (g of protein + g of lipid + g of ash). On the basis of Atwater’s constant, total energy was calculated as follows: energy (kcal/g) = 5.65 × (g of protein) + 4.1 × (g of carbohydrate) + 9.3 × (g of lipid) [44].

### 2.3. Sugar Analysis

The sugars profile of sucrose, fructose, glucose, maltose, and lactose of bee pollen were analyzed by high performance liquid chromatography with an evaporative light scattering detector (1260 Infinity II, Agilent, Waldbronn, Germany) according to the methods of Yang et al. and the Taiwan Food and Drug Administration [45,46]. For sugar extraction, bee pollen (1 g) was ground in liquid nitrogen. The pollen powder was ultrasonicated with 20 mL 50% ethanol solution for 20 min, the shaken for 10 min and centrifuged for 10 min. The supernatant was filtered through 0.22-μm filter paper, and 10 μL of the filtered sample was loaded into the ZORBAX NH_2_ column (250 mm × 4.6 mm i.d., 5 μm, Agilent, Santa Clara, CA, USA) with 75% acetonitrile as the mobile phase at a flow rate of 1.0 mL/min with an oven temperature of 30 °C. The drift tube temperature was set at 80 °C. N_2_ served as the carrier gas at a flow of 2.0 mL/min. The sugar peaks were identified by comparing the retention times obtained from the reference standards. The sugar content in bee pollen was calculated according to the standard curves made by each reference sugar (Sigma-Aldrich, St. Louis, MO, USA). The results are reported as grams of sugar per 100 g of dry bee pollen. The calibration parameters of sugar standards, such as linearity range, *R*^2^, and limit of quantification (LOQ) are shown below. The linearity of different concentration range for the sugars is 0.03~0.20 g/100 mL (*R*^2^ ≥ 0.995). The LOQ for sugars is 0.3 g per 100 g of dry bee pollen.

### 2.4. Amino Acid Analysis

For total amino acid analysis, bee pollen (100 mg) defatted with petroleum ether was hydrolyzed with 1 mL of 4 N CH₃SO₃H containing 0.2% tryptamine for 72 h at 115 °C in 25-mL vacuum-sealed ampules [47], except that tryptophan was determined by hydrolyzing bee pollen with 10 mL of 4.2 M NaOH containing three drops of 1-octanol for 72 h at 110 °C (AOAC method 988.15). The pH of the cooled and filtered hydrolysate was adjusted and then analyzed using a reverse-phased liquid chromatography equipped with a UV detector (1100 series, Agilent). Eighteen amino acids were quantified by comparing peak areas with those of standard amino acids (Sigma-Aldrich, Buchs, Switzerland). The results are reported as grams of amino acid per 100 g of dry bee pollen. The calibration parameters of amino acid standards are shown below. The linearity of different concentration ranges for the amino acids is 60~2500 pmol/μL, except for cystine and tryptophan, which used 30~1000 pmol/μL (*R*^2^ ≥ 0.995). The LOQ for amino acids is 2.0 mg per 100 g of dry bee pollen.

The chemical scores of the EAAs of bee pollen were used to evaluate the nutritive value of bee pollen. It was calculated by comparing the amino acids of bee pollen with the minimum requirements of honey bees and humans, i.e., the ratio of the amount of the amino acid in bee pollen to the same amount of the corresponding amino acid in the minimum requirements of honey bees and humans multiplied by 100 [16,20,48,49].

### 2.5. Fatty Acid Analysis

To determine the fatty acid composition in bee pollen, we derivatized the fatty acids of bee pollen to produce fatty methyl esters (FAMEs) according to the methods reported by Lepage and Roy [50] and the World Health Organization [51]. FAME analysis was performed using gas chromatography equipped with a flame ionization detector (6890, Agilent) [52]. Fat was obtained by Soxhlet extracting bee pollen (200 mg) with ether containing 100 mg pytogallic acid for 50 min at 85 °C. The solvent was removed in a rotary evaporator at 40 °C and redissolved in *n*-hexane. Subsequently, FAMEs were prepared as follows: 1 mL oil sample was mixed with 1 mL 1 N NaOH–methanol complex solution for 15 min at 80 °C; 1 mL 14% boron trifluoride–methanol complex solution was then added and incubated for 15 min at 110 °C; and finally, 1 mL *n*-hexane and 6 mL saturated NaOH solution were added. After incubation for 30 min at room temperature, the upper layer was collected and filtered for gas chromatography analysis. Triheneicosanoin (C_21:0_ TAG) was served as internal standard (Nu-Chek Prep, Elysian, MN, USA). Separation was performed using a HP- 88 fused capillary column (100 m × 0.25 mm i.d., 0.2 μm, Agilent). The injector temperature was set at 250 °C, and the detector temperature was set at 300 °C; He served as the carrier gas at a flow of 0.5 mL/min. The column was initially operated at 170 °C, which was maintained for 40 min, then increased to 200 °C at 3 °C/min, and finally maintained for 50 min. The split ratio was 1:40, and the injected volume was 1.0 μL. FAMEs were identified and quantified by comparing peak areas with those of standard FAME mixtures (Nu-Chek Prep). The amounts of individual FAMEs (W_FAMEx_) were calculated using the expression W_FAMEx_ = (A_x_ × W_IS_ × 1.0040 × R_x_)/A_IS_, where A_x_ is peak area counts for FAME *x* in test sample, W_IS_ is weight of the internal standard (C_21:0_ TAG), 1.0040 is the conversion coefficient of the internal standard (C_21:0_ TAG) from TAG to FAME, A_IS_ is peak area counts of the C_21:0_ FAME, and R_x_ is the theoretical flame ionization detector correction factor for FAMEs relative to the C_21:0_ FAME [51]. The results are expressed as the percentage of total FAMEs. The LOQ for each FAMEs is 0.01%.

### 2.6. Statistical Analysis

All chemical analyses were carried out in three technical repetitions. All statistical analyses were performed using SAS version 9.4 (SAS Institute Inc., Cary, NC, USA). Data of samples from three places are expressed as the mean ± standard deviation (SD). A nonparametric Kruskal–Wallis test was used to compare the contents of nutrients among bee pollen samples. In contrast, the Mann–Whitney test was only used to compare Eicosatrienoic acid (C_20:3_) content among bee pollen samples. The Nemenyi post-hoc test was used for multiple pairwise comparisons when the Kruskal–Wallis test revealed significant differences. Data of samples from a single place are expressed as the mean value of the three technical repetitions.

## 3. Results and Discussion

### 3.1. Bee Pollen Samples

**Figure 1:**
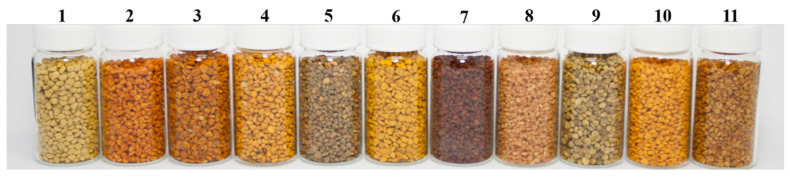
Eleven monofloral bee pollen samples. (1) *Brassica napus*, (2) *Bidens pilosa* var. *radiata*, (3) *Camellia sinensis*, (4) *Fraxinus griffithii*, (5) *Prunus mume*, (6) *Rhus chinensis* var. *roxburghii*, (7) *Bombax ceiba*, (8) *Hylocereus costaricensis*, (9) *Liquidambar formosana*, (10) *Nelumbo nucifera*, and (11) *Zea mays*.

Many studies have worked on the chemical composition of bee pollen. The results of more than 100 bee pollens revealed that the nutritional value of bee pollen varies depending on its floral origin [3,6,7,8,14,15,16,20,21,53,54]. In this study, we expanded the nutritional information of bee pollen by investigating 11 bee pollen samples collected in Taiwan (Figure 1). Detailed information regarding the 11 plants for bee pollen is listed in Table 1, and the pictures of the flowers are shown in Supplementary Figure S1. Tea tree (Cs), Japanese apricot (Pm), pitaya (Hc), lotus (Nn), and corn (Zm) are popular crop plants in Taiwan and account for an annual bee pollen yield of at least 200 tons. Regarding non-crop plants, rape (Bn), beggartick (Bp), evergreen ash (Fg), nutgall tree (Rc), red cotton tree (Bc), and Formosan gum (Lf) are common plants, with a wide distribution in Taiwan. They can yield approximately 250 tons in total. However, the yield of bee pollen has largely varied recently due to dramatic climate changes. Four of the 11 bee pollen samples (i.e., Bn, Cs, Nn, and Zm) have been studied previously [16,20]. However, the effect of different geographical locations on the nutritive value of bee pollen can be determined through their simultaneous comparison. The nutritive value of the remaining seven bee pollen samples is presented for the first time in this paper. The usefulness of bee pollen obtained from different floral origins for bees and humans can be assessed based on its protein, amino acid, and fatty acid compositions.

### 3.2. Proximate Analysis of Bee Pollen

Table 2 summarized the findings of the proximate analysis of the 11 bee pollen samples. In the 11 bee pollen samples, carbohydrate was the predominant macronutrient, followed by proteins and lipids. Carbohydrates range widely from 60.4% in Bc to 78.8% in Bp. The values were similar to the findings by Yang et al. [16] but were higher than those references summarized by Campos et al. who showed 13–55% [12]. The big difference is probably caused by different analytic methods. Campos et al. stated that the carbohydrate content determined by HPLC methods would be lesser than the calculated carbohydrate content (100 minus the sum of lipid, protein, and ash) which would contain crude fiber and cell wall material [12]. The bee pollen samples Bp, Zm, Lf, Fg, and Nn had higher carbohydrate contents than the average carbohydrate content of the 11 bee pollen samples (68.6 %).

The protein content of bee pollen is the most crucial characteristic and is mainly used to classify the quality of bee pollen as follows: excellent (>25%), average (20–25%), and poor (<20%) [14]. The protein content of the 11 bee pollen samples considerably varied between 15.9% in Lf and 32.2% in Bc, with an average value of 24.4%. The bee pollen samples Bc, Cs, Pm, Hc, Bn, and Rc; Fg; and Bp, Lf, Nn, and Zm were determined to have excellent, average, and poor quality, respectively. The lipid content of the 11 bee pollen samples ranged from 2.0% in Rc to 8.8% in Hc, with a mean value of 4.2%. This value was fitted into the 1–13% lipid range from references summarized by Campos et al. [12]. The bee pollen samples Hc, Bn, Lf, Nn, and Bc have higher than 4.2% lipid content. Generally, the average water and ash contents of the 11 bee pollen samples were 19.0% and 2.8%, respectively. The values of water and ash in bee pollen samples of this study is close to the values obtained from other bee pollen samples [12,16]. Sometimes, beekeepers in Taiwan obtained high moisture in fresh bee pollen samples (more than 20%), which was presumed to be due to the high humidity of the harvest sites in mountainous areas. The energy value of the pollen samples ranged from 443.4 to 488.6 kcal/100 g dry mass. Accordingly, bee pollen was recommended as part of a low-calorie diet.

Monofloral bee pollen with different geographic origins exhibit considerable variations in protein and fatty acid contents. For example, the protein content of Bn varies as follows: 18.9% (Saudi Arabia), 19.6% (India), 23.0% (Brazil), 22.8–26.1% (Australia), and 27.3% (China), and the lipid content in Bn varies as follows: 4.7% (Brazil), 6.6% (China), and 12.4% (India) [14,16,20,55,56]. A similar trend has been found for other bee pollen samples, including Cs, Nn, and Zm. Regarding the bee pollen Zm, the protein content of Taiwan Zm is similar to that of China Zm (17.9%) and is higher than that of Australia Zm and Greece Zm (14.9%) [14,15,16]. The carbohydrate content (>75%) of Taiwan Zm and China Zm is similar. However, the lipid content of China Zm (4.0%) is higher than that of Taiwan Zm (2.8%) [16]. Regarding the bee pollen samples Cs and Nn, we compared our results with previous results from China and found a similar macronutrient content between Taiwan and China, but China Cs had a higher lipid content (5.25%), and China Nn had a lower protein content (17%) [16].

### 3.3. Sugar Analysis of Bee Pollen

Table 3 presents the total sugar content in the 11 bee pollen samples. The sugar content ranged from 25.2% in Hc to 44.8% in Lf, with a mean value of 36.7%. The predominant sugars in the 11 bee pollen samples were sucrose, glucose, and fructose, with mean values of 2.5%, 13.6%, and 18.8%, respectively, and they accounted for approximately 95% of the total sugar in the 11 bee pollen samples. This value is consistent with other bee pollen studies [12]. The reducing sugar content was the highest in Nn (38%) and the lowest in pollen Hc (24.4%). The reducing sugar content of bee pollen is associated with the presence of plant nectar/bee saliva, which is commonly used by bees as glue for making the bee pollen pellet [12].

### 3.4. Amino Acid Analysis

The contents of 18 amino acids (i.e., 10 EAAs and 8 nonessential amino acids [NEAAs]) in the 11 bee pollen samples were investigated and are presented in Table 4. The average value of the 18 amino acids of the 11 bee pollen samples was 20.38 g/100 g dry mass; the mean value of each amino acid was 1.13 g/100 g dry mass. The amino acids leucine (mean value of 1.62 g/100 g dry mass), lysine (mean value of 1.51 g/100 g dry mass), valine (mean value of 1.25 g/100 g dry mass), alanine (mean value of 1.27 g/100 g dry mass), aspartic acid (mean value of 2.47 g/100 g dry mass), glutamic acid (mean value of 2.47 g/100 g dry mass), and proline (mean value of 1.7 g/100 g dry mass) were recognized to be predominant in bee pollen because their mean values were >1.13 g/100 g dry mass. The high contents of the predominant amino acids could be detected in the bee pollen samples Bn, Cs, Pm, Rc, Bc, and Hc. However, all the 11 bee pollen samples contained low concentrations of methionine, tryptophan, and cystine. The mean value was below 0.5 g/100 g dry mass. Many studies have reported that tryptophan and methionine are limiting amino acids in bee pollen [16,20,54,57]. EAA and NEAA contents were higher than the mean value (9.62 g/100 g dry mass and 10.76 g/100 g dry mass, respectively) in the bee pollen samples Bn, Cs, Pm, Rc, Bc, and Hc.

The nutritive value of proteins for any biological function is limited by the relative proportions of comprising EAAs [24]. High levels of EAAs can provide a high nutritional value for honey bees and humans [18,24,25]. Therefore, in reference to the minimal amino acid requirements of honey bees and humans [24,25], the chemical scores of the EAAs of bee pollen can be calculated, reflecting the nutritional value of bee pollen for bees and humans. As shown in Supplementary Table S1, the chemical scores of EAAs in the 11 bee pollen samples for bees could be arranged in descending order as follows: Hc (186.27) > Nn (175.50) > Bp (174.11) > Cs (163.20) > Fg (163.10) > Rc (162.43) > Bn (160.58) > Bc (157.72) > Lf (149.52) > Pm (147.61) > Zm (140.32). According to the chemical scores of EAAs, only the EAA content of the bee pollen Hc met the requirements of honey bees (Supplementary Table S1). However, the bee pollen samples Bn, Bp, Cs, and Rc only had one limiting amino acid (methionine), and the bee pollen Nn consisted of the limiting amino acid tryptophan. In addition, the two limiting amino acids methionine and tryptophan were found in the bee pollen samples Fg, Pm, and Bc. Furthermore, three limiting amino acids were found in the bee pollen samples Lf (histidine, tryptophan, and valine) and Zm (isoleucine, methionine, and tryptophan).

In terms of the minimal amino acid requirements of adult humans, the chemical scores of EAAs in the 11 bee pollen samples can be arranged in descending order as follows: Hc (162.91) > Bp (156.22) > Nn (152.60) > Rc (151.29) > Cs (142.05) > Fg (141.12) > Bn (137.43) > Bc (135.63) > Pm (126.39) > Zm (118.83) > Lf (114.65) (Supplementary Table S2). The 11 bee pollen samples nearly satisfied the level of all determined EAAs, as recommended by FAO/WHO/UNU, 2007 consultation [25]. The content of EAAs of the bee pollen Hc met the requirements for humans (Supplementary Table S2). The bee pollen samples Bn, Bp, Cs, Fg, Pm, Rc, Bc, and Nn had one limiting amino acid, namely methionine. The bee pollen Zm had two limiting amino acids, namely methionine and tryptophan, and the bee pollen Lf had three limiting amino acids, namely histidine, tryptophan, and valine. From the viewpoint of human nutrition, bee pollen can be a favorable nutrition source of lysine and threonine, which are limited in daily diet cereals [25].

Compared with results reported by Taha et al. (2019) and Yang et al. (2013) who characterized 5 and 12 bee pollen samples in Saudi Arabia and China, respectively, we examined the amino acid contents of the bee pollen samples Bn, Cs, Nn, and Zm in this study [16,20]. The amino acid contents of four bee pollen samples in Taiwan are similar to those of four bee pollen samples in China, except that the tryptophan content was higher in the Chinese bee pollen [16]. However, amino acid contents vary in Bn from Taiwan and Saudi Arabia. For example, the mean chemical score of bee pollen for bees in Taiwan and Saudi Arabia is 161 and 102, respectively [20]. The chemical scores for the EAAs arginine, histidine, isoleucine, leucine, lysine, methionine, phenylalanine, threonine, tryptophan, and valine of Bn in Saudi Arabia are 69.1, 140.7, 79.5, 128.0, 135.6, 17.7, 106.8, 154.8, 63.0, and 127.8. This is different from our study, and the different amino acid contents of Bn from Saudi Arabia and Taiwan are assumed to be caused by geographical factors.

### 3.5. Fatty Acid Analysis

Table 5 presents fatty acid profiles determined for the 11 bee pollen samples. We found that 16 fatty acids accounted for 90.18%, 92.99%, 96.25%, 87.83%, 95.97%, 86.25%, 89.45%, 96.54%, 98.48%, 98.0%, and 92.05% of total fatty acids in the bee pollen samples Bn, Bp, Cs, Fg, Pm, Rc, Bc, Hc, Lf, Nn, and Zm, respectively. Five of the 16 fatty acids, namely palmitic acid (C_16:0_), stearic acid (C_18:0_), oleic acid (C_18:1_), linoleic acid (C_18:2_), and linolenic acid (C_18:3_), were dominant fatty acids (DFAs) in all bee pollen samples and exhibited high levels. The total contents of the five DFAs in the 11 bee pollen samples ranged widely from 65.96% to 97.44% and could be ranked as follows: Lf (97.44%) > Nn (95.0%) > Hc (91.58%) > Zm (89.48%) > Pm (83.79%) > Bc (83.1%) > Fg (81.14%) > Bn (79.25%) > Rc (78.43%) > Cs (77.96%) > Bp (65.96%). Among the five DFAs, the highest content of palmitic acid (56.0 %) was obtained from the bee pollen Nn, stearic acid (10.25%) from the bee pollen Bp, oleic acid (47.5%) from the bee pollen Fg, linoleic acid (37.2%) from the bee pollen Lf, and linolenic acid (45.6%) from the bee pollen Hc.

The remaining 11 fatty acids are classified as nondominant fatty acids (NDFAs), which only appear at low concentrations in some bee pollen samples. The total contents of 11 NDFAs in the 11 bee pollen samples ranged widely from 1.0% to 27.03 %. The diverse NDFA content was found in the bee pollen samples Bn, Bp, Pm, Rc, Bc, Lf, and Zm, with more than eight fatty acids. In terms of the pollen samples with an NDFA concentration of >1%, Bp contained five NDFAs, namely caproic acid (7.27%), myristic acid (2.37%), eicosenoic acid (1.63%), eicosadienoic acid (12.46%), and lignoceric acid (1.0%). Pm contained four NDFAs, namely pentadecanoic acid (5.15%), arachidic acid (1.13%), eicosatrienoic acid (1.79%), and lignoceric acid (1.18%). Cs contained three NDFAs, namely behenic acid (5.87%), lignoceric acid (3.64%), and nervonic acid (1.81%). Two NDFAs with concentrations of >1% were found in the bee pollen samples Bn, Fg, Rc, Bc, and Hc: myristic acid (6.45%) and arachidic acid (1.67%) in the bee pollen Bn; myristic acid (1.19%) and eicosenoic acid (2.72%) in the bee pollen Fg; arachidic acid (2.04%) and lignoceric acid (1.62%) in the bee pollen Rc; eicosenoic acid (1.33%) and erucic acid (2.38%) in the bee pollen Bc; and behenic acid (1.03%) and lignoceric acid (1.78%) in the bee pollen Hc. Moreover, the bee pollen samples Lf and Zm contain 10 and 11 NDFAs, respectively, but the concentrations of these NDFAs were <1%. The bee pollen Nn contained few NDFAs, only containing myristic acid, eicosatrienoic acid, and lignoceric acid, which accounted for 1% of total, respectively.

Compared with results reported by Yang et al. (2013) (who characterized fatty acid profiles in the bee pollen samples Bn, Cs, Nn, and Zm), similar DFA contents were observed in four bee pollen samples from both places, except that China Cs and Zm had higher linolenic acid contents of 46.9% and 52.0%, respectively [16]. The same bee pollen obtained from different geographic origins showed different fatty acid proportions. For example, bee pollen Zm from China contained two DFAs: palmitic acid (25.1%) and linolenic acid (52.0%); however, Zm from Egypt contained high contents of oleic acid (42.0%) and myristic acid (40.0%) [16,58]. Similarly, myristic acid (20.7%) and linolenic acid (30.8%) were present in bee pollen Bn from China. However, Bn from India showed high contents of linolenic acid (29.1%) and eicosatrienoic acid (13.8%) [16,55]. The different fatty acid profiles in bee pollen might be caused by various environmental conditions, such as soil composition, atmospheric conditions, and other factors that affect plant growth [59,60].

Both linoleic acid and linolenic acid have been linked to learning behavior and brood development in honey bees [28,29,30,31]. They have also been demonstrated to have bactericidal and antifungal activities, which can protect bees against pathogens [26]. In humans, both fatty acids play key roles in regulating activities related to homeostasis, such as insulin activity and cardiovascular and immune responses [61,62,63]. In addition, bee pollen with high contents of oleic and palmitic acids have crucial roles in bee nutrition [26]. Furthermore, stearic acid is the substrate involved in the biosynthesis of 9-oxo-2-decenoic acid in queen bees (queen pheromone) and of 10-hydroxy-2-decenoic acid, which accounts for 60–80% of the total fatty acid composition in the royal jelly produced by bee workers [64,65]. In humans, the DFAs palmitic acid, stearic acid, and oleic acid play crucial roles in maintaining normal multiple physiological activities [66,67,68]. Therefore, the contents of DFAs including PUFAs can also be regarded as a nutritional index of bee pollen.

## 4. Conclusions

Bee pollen has long been regarded as a natural product with high nutritional value. However, the nutritional contents of bee pollen vary considerably with floral species and geographic origins. The continuous accumulation of this pollen nutrition information helps determine the appropriate bee pollen for food supplements for animals or humans. Summarizing the data we obtained, the average carbohydrate, protein, and lipid contents of the 11 bee pollen samples is 68.6%, 24.4%, and 4.2%, respectively. The bee pollen samples Bn, Cs, Pm, Rc, Bc, and Hc, had excellent protein contents, which accounted for >25%. The amino acids leucine, lysine, valine, alanine, aspartic acid, glutamic acid, and proline are dominant amino acids in bee pollen, and each of them contains more than 1.25 g/100 g dry mass. On the basis of the minimal amino acid requirements of honey bees and humans, the top six bee pollen samples were Hc, Nn, Bp, Cs, Fg, and Rc. Regarding fatty acid profile in the 11 bee pollen samples, palmitic acid, stearic acid, oleic acid, linoleic acid, and linolenic acid, were dominant fatty acids. The content of the five dominant fatty acids ranged widely between 65.96% and 97.44% in the 11 bee pollen samples. In light of the above findings, we would be able to evaluate the nutritional values of 11 bee pollens and contribute to a database of food composition. Additionally, these data can be used to guide recommendations made by health agencies and choices made by consumers as well as the production of bee pollen by beekeepers. Further intensive research, e.g., regarding the content of phytometabolites (carotenoids, phenols, flavonoids, and vitamins) or pollen-related allergen and pesticide residues, will be conducted to enrich the knowledge of bee pollen for promoting its application in the food industry.

## Figures and Tables

**Table 1:** Common botanical origins for bee pollen production in Taiwan.

Item	Species	Common Name	Foodstuff	Cultivation	Characteristic	Bloom Time
1	*Brassica napus*	Rape	Non-crop	Cultivate	Herbaceous	Dec.–Jan. ^†^
2	*Bidens pilosa* var. *radiata*	Beggartick	Non-crop	Wild	Herbaceous	Jan.–Dec.
3	*Camellia sinensis*	Tea tree	Crop	Cultivate	Woody	Oct.–May
4	*Fraxinus griffithii*	Evergreen ash	Non-crop	Wild	Woody	May–Jun.
5	*Prunus mume*	Japanese apricot	Crop	Cultivate	Woody	Jan.
6	*Rhus chinensis* var. *roxburghii*	Nutgall tree	Non-crop	Wild	Woody	Sep.–Oct.
7	*Bombax ceiba*	Red cotton tree	Non-crop	Cultivate	Woody	Mar.–Apr.
8	*Hylocereus costaricensis*	Pitaya	Crop	Cultivate	Herbaceous	Apr.–Oct.
9	*Liquidambar formosana*	Formosan gum	Non-crop	Cultivate/wild	Woody	Mar.–Apr.
10	*Nelumbo nucifera*	Lotus	Crop	Cultivate	Herbaceous	May–Jun.
11	*Zea mays*	Corn	Crop	Cultivate	Herbaceous	Dec.–Jan. ^†^

^†^ The period refers to the bloom time under regular condition of intensive cultivation.

**Table 2:** Proximate composition and energy value of bee pollen samples (g/100 g dry mass) ^†^.

Component	Each Pollen Sample from Three Places						Each Pollen Sample from Single Place					Average
	Bn	Bp	Cs	Fg	Pm	Rc	Bc	Hc	Lf	Nn	Zm	
Carbohydrate	62.6 ± 2.3 b	78.8 ± 1.0 a	63.7 ± 3.8 ab	73.0 ± 1.2 ab	64.9 ± 3.0 ab	67.4 ± 0.9 ab	60.4	60.9	74.8	71.8	78.1	68.6
Protein	27.2 ±1.3 ns	16.4 ± 0.5 ns	29.9 ± 3.4 ns	20.8 ± 0.9 ns	28.3 ± 2.8 ns	27.0 ± 0.7 ns	32.2	27.8	15.9	18.4	17.2	24.4
Lipid	7.1 ± 1.1 a	3.2 ± 0.6 ab	3.1 ± 0.3 ab	4.1 ± 0.5 ab	3.6 ± 0.2 ab	2.0 ± 0.1 b	4.2	8.8	7.0	5.3	2.8	4.2
Ash	3.1 ± 0.2 ab	1.7 ± 0.1 b	3.2 ± 0.2 ab	2.0 ± 0.1 ab	3.2 ± 0.2 ab	3.5 ± 0.1 a	3.2	2.5	2.3	4.5	2.0	2.8
Energy ^‡^	476.1	445.1	459.3	455.1	459.8	448.4	468.6	488.6	461.6	447.6	443.4	458.3
Water content ^£^	20.7 ± 1.2	19.2 ± 2.0	22.2 ± 0.7	12.8 ^¤^	20.4 ^¤^	19.4 ^¤^	18.2	19.5	18.3	5.3	21.9	19.0

^†^ Means with different letters within the same row are significantly different (*p* < 0.05); ns: not statistically significant. ^‡^ Values are expressed as kcal/100 g dry mass. ^£^ Values are calculated from fresh bee pollen samples (g/100 g wet mass). ^¤^ For water content measurement of bee pollen Fg, Pm, and Rc, only 1, 2, and 2 fresh bee pollen samples were used for calculation, respectively, excluding the four pre-dried samples (2 of Fg, 1 of Pm, and 1 of Rc).

**Table 3:** Sugar composition of bee pollen samples (g/100 g dry mass) ^†^.

Sugar	Each Pollen Sample from Three Places						Each Pollen Sample from Single Place					Average
	Bn	Bp	Cs	Fg	Pm	Rc	Bc	Hc	Lf	Nn	Zm	
Total sugar ^‡^	33.1 ± 4.1 ns	43.6 ± 2.5 ns	32.1 ± 6.2 ns	43.6 ± 5.5 ns	33.9 ± 3.0 ns	32.6 ± 0.8 ns	39.3	25.2	44.8	44.7	34.0	36.7
Sucrose	1.2 ± 0.8 ns	2.0 ± 0.5 ns	0.7 ± 0.4 ns	5.5 ± 0.9 ns	1.0 ± 0.7 ns	2.8 ± 3.3 ^¤^	5.2	ND ^¥^	6.2	5.5	ND	2.5
Glucose	11.9 ± 3.0 ab	18.6 ± 1.5 a	10.3 ± 2.7 ab	16.8 ± 1.5 ab	12.6 ± 1.8 ab	10.2 ± 0.6 b	14.7	8.7	16.3	17.3	13.9	13.6
Fructose	18.8 ± 1.4 ns	17.8 ± 0.8 ns	18.9 ± 1.9 ns	20.3 ± 3.0 ns	18.2 ± 1.6 ns	18.4 ± 2.3 ns	18.8	15.7	20.5	20.7	18.3	18.8
S+G+F	31.9	38.4	29.9	42.5	31.8	31.5	38.7	24.4	43.0	43.5	32.2	34.8
(S+G+F)/T ^£^ (%)	96.6	88.0	93.0	97.6	94.0	96.7	98.6	97.1	95.9	97.3	94.8	94.9

^†^ Means with different letters within the same row are significantly different (*p* < 0.05); ns: not statistically significant. ^‡^ The sum of sucrose, glucose, fructose, maltose, and lactose. ^£^ S, sucrose. G, glucose. F, fructose. T, total sugar. ^¤^ Results are not included in analysis of variance due to excessive standard deviation. ^¥^ ND, not detected.

**Table 4:** Amino acid composition of bee pollen samples (g/100 g dry mass) ^†^.

Amino Acid	Each Pollen Sample from Three Places						Each Pollen Sample from Single Place					Average
	Bn	Bp	Cs	Fg	Pm	Rc	Bc	Hc	Lf	Nn	Zm	
EAA ^‡^												
Arginine	1.30 ± 0.12 ab	0.57 ± 0.05 b	1.36 ± 0.13 a	0.91 ± 0.03 ab	1.23 ± 0.14 ab	1.05 ± 0.06 ab	1.45	1.40	1.02	0.94	0.74	1.08
Histidine	0.46 ± 0.14 ns	0.73 ± 0.07 ns	0.74 ± 0.17 ns	0.67 ± 0.24 ns	0.58 ± 0.13 ns	0.54 ± 0.06 ns	0.80	0.61	0.16	0.50	0.30	0.59
Isoleucine	1.21 ± 0.11 ab	0.69 ± 0.06 b	1.36 ± 0.24 a	0.95 ± 0.04 ab	1.15 ± 0.20 ab	1.12 ± 0.04 ab	1.50	1.50	0.69	0.96	0.67	1.08
Leucine	1.82 ± 0.15 ab	1.06 ± 0.08 b	2.07 ± 0.28 a	1.42 ± 0.06 ab	1.77 ± 0.30 ab	1.69 ± 0.06 ab	2.05	2.12	1.24	1.34	1.03	1.62
Lysine	1.91 ± 0.25 a	1.00 ± 0.10 b	1.76 ± 0.18 ab	1.21 ± 0.11 ab	1.61 ± 0.32 ab	1.66 ± 0.13 ab	1.81	2.05	1.25	1.28	1.00	1.51
Methionine	0.35 ± 0.05 ns	0.21 ± 0.14 ns	0.40 ± 0.06 ns	0.29 ± 0.22 ns	0.28 ± 0.12 ns	0.35 ± 0.16 ns	0.48	0.46	0.26	0.28	0.12	0.32
Phenylalanine	1.11 ± 0.17 ns	0.80 ± 0.12 ns	1.32 ± 0.24 ns	0.89 ± 0.05 ns	1.14 ± 0.25 ns	1.13 ± 0.09 ns	1.29	1.38	0.58	0.79	0.58	1.03
Threonine	1.06 ± 0.08 ns	0.57 ± 0.06 ns	1.07 ± 0.12 ns	0.76 ± 0.07 ns	0.99 ± 0.16 ns	0.96 ± 0.06 ns	1.19	1.12	0.51	0.73	0.63	0.89
Tryptophan	0.28 ± 0.04 ns	0.18 ± 0.07 ns	0.30 ± 0.01 ns	0.13 ± 0.07 ns	0.22 ± 0.05 ns	0.51 ± 0.39 ns	0.20	0.35	0.09	0.14	0.10	0.25
Valine	1.31 ± 0.04 ab	0.84 ± 0.08 b	1.56 ± 0.15 a	1.10 ± 0.04 ab	1.44 ± 0.18 ab	1.34 ± 0.08 ab	1.79	1.85	0.29	1.08	0.99	1.25
Total EAA	10.81 ± 0.61	6.66 ± 0.61	11.96 ± 1.41	8.33 ± 0.65	10.40 ± 1.79	10.35 ± 0.88	12.58	12.84	6.09	8.04	6.17	9.62
NEAA ^£^												
Alanine	1.42 ± 0.09 ab	0.88 ± 0.07 b	1.60 ± 0.20 a	1.11 ± 0.04 ab	1.45 ± 0.21 ab	1.30 ± 0.08 ab	1.56	1.73	0.66	1.00	0.99	1.27
Aspartic acid	2.56 ± 0.22 ab	1.45 ± 0.10 b	2.70 ± 0.30 ab	2.64 ± 0.12 ab	3.14 ± 0.50 a	2.43 ± 0.24 ab	3.24	3.19	1.68	2.31	1.57	2.47
Cystine	0.37 ± 0.04 a	0.69 ± 0.87 ^¥^	0.24 ± 0.11 ab	0.12 ± 0.04 b	0.26 ± 0.27 ^¥^	0.18 ± 0.03 ab	0.14	0.29	0.21	0.03	0.15	0.28
Glutamic acid	2.64 ± 0.16 ab	1.46 ± 0.15 b	3.04 ± 0.35 a	2.08 ± 0.09 ab	2.88 ± 0.43 ab	2.74 ± 0.18 ab	3.33	3.48	1.73	2.16	1.64	2.47
Glycine	1.21 ± 0.09 ab	0.73 ± 0.05 b	1.31 ± 0.16 a	0.95 ± 0.06 ab	1.19 ± 0.14 ab	1.14 ± 0.07 ab	1.42	1.31	0.58	0.82	0.78	1.06
Proline	1.24 ± 0.12 ab	0.63 ± 0.10 b	2.31 ± 0.76 ab	1.09 ± 0.18 ab	2.47 ± 0.29 ab	3.35 ± 0.86 a	1.73	0.98	0.75	0.55	1.99	1.70
Serine	1.10 ± 0.05 ab	0.63 ± 0.11 b	1.22 ± 0.12 a	0.88 ± 0.07 ab	1.03 ± 0.22 ab	1.02 ± 0.10 ab	1.19	1.07	0.58	0.80	0.70	0.96
Tyrosine	0.57 ± 0.07 ns	0.35 ± 0.04 ns	0.65 ± 0.05 ns	0.51 ± 0.12 ns	0.64 ± 0.12 ns	0.64 ± 0.14 ns	0.73	0.65	0.18	0.56	0.37	0.55
Total NEAA	11.10 ± 0.57	6.81 ± 0.49	13.07 ± 1.74	9.37 ± 0.46	13.05 ± 1.6	12.80 ± 0.37	13.34	12.70	6.36	8.24	8.20	10.76
TAA ^¤^	21.91 ± 1.17	13.47 ± 0.37	25.02 ± 3.11	17.71 ± 1.11	23.46 ± 3.39	23.15 ± 1.21	25.91	25.54	12.45	16.27	14.37	20.38
TEAA/TAA (%)	49.34 ± 0.20	49.39 ± 3.88	47.80 ± 0.99	47.04 ± 0.74	44.24 ± 1.16	44.66 ± 1.57	48.53	50.27	48.90	49.39	42.96	47.28

^†^ Means with different letters within the same row are significantly different (*p* < 0.05); ns: not statistically significant. ^‡^ EAA, essential amino acids. ^£^ NEAA, nonessential amino acids. ^¤^ TAA, total amino acids. ^¥^ Results are not included in analysis of variance due to excessive standard deviation.

**Table 5:** Fatty acid composition of bee pollen samples ^†^.

Fatty Acid	Each Pollen Sample from Three Places						Each Pollen Sample from Single Place					Average
	Bn	Bp	Cs	Fg	Pm	Rc	Bc	Hc	Lf	Nn	Zm	
DFA ^‡^												
Palmitic acid; C_16:0_	19.42 ± 2.54 ab	18.34 ± 0.93 ab	25.45 ± 2.36 a	10.06 ± 4.33 b	14.02 ± 1.99 ab	15.64 ± 2.50 ab	14.56	25.74	23.40	56.00	38.44	20.30
Stearic acid; C_18:0_	5.70 ± 0.67 ab	10.25 ± 1.26 a	3.65 ± 0.23 ab	9.54 ± 6.50 ab	1.84 ± 0.30 b	2.79 ± 0.52 ab	1.83	1.88	2.35	3.00	1.30	4.85
Oleic acid; C_18:1_	10.55 ± 1.25 ab	6.30 ± 2.52 b	13.17 ± 3.51 ab	47.50 ± 11.18 a	11.68 ± 0.69 ab	25.63 ± 4.23 ab	19.19	4.26	26.62	17.00	7.95	18.24
Linoleic acid; C_18:2_	5.15 ± 0.3 b	12.24 ± 0.23 ab	6.01 ± 0.31 ab	8.50 ± 8.99 ^¤^	34.61 ± 1.62 a	12.08 ± 2.16 ab	31.00	14.10	37.20	9.00	8.58	14.59
Linolenic acid; C_18:3_	38.43 ± 3.36 a	18.84 ± 3.22 ab	29.68 ± 6.20 ab	5.55 ± 2.16 b	21.64 ± 1.72 ab	22.30 ± 5.48 ab	16.50	45.60	7.90	10.00	33.21	22.72
Total DFA	79.25 ± 4.97	65.96 ± 1.44	77.96 ± 3.11	81.14 ± 11.88	83.79 ± 4.24	78.43 ± 5.43	83.10	91.58	97.44	95.00	89.48	80.70
NDFA ^£^												
Caproic acid; C_6:0_	0.37 ± 0.07 ab	7.27 ± 0.36 a	ND ^¥^	ND	0.14 ± 0.12 ab	0.07 ± 0.06 b	ND	ND	ND	ND	0.17	1.03
Myristic acid; C_14:0_	6.45 ± 0.90 a	2.37 ± 0.18 ab	0.78 ± 0.14 ab	1.19 ± 0.94 ab	0.39 ± 0.29 b	0.63 ± 0.18 ab	0.41	0.97	0.14	1.00	0.20	1.66
Pentadecanoic acid; C_15:0_	0.10 ± 0.02 ns	0.21 ± 0.02 ns	ND	ND	5.15 ± 4.47 ns	0.09 ± 0.08 ns	ND	ND	0.03	ND	0.07	0.73
Arachidic acid; C_20:0_	1.67 ± 1.30 ns	ND	ND	0.65 ± 0.09 ns	1.13 ± 0.98 ns	2.04 ± 1.86 ns	0.38	0.81	ND	ND	ND	0.77
Eicosenoic acid; C_20:1_	0.27 ± 0.20 b	1.63 ± 0.12 ab	0.97 ± 0.06 ab	2.72 ± 1.43 a	0.56 ± 0.24 ab	0.79 ± 0.34 ab	1.33	ND	0.19	ND	0.12	0.98
Eicosadienoic acid; C_20:2_	0.20 ± 0.03 b	12.46 ± 0.65 a	0.32 ± 0.20 ab	ND	0.33 ± 0.18 ab	0.73 ± 0.29 ab	0.18	0.22	0.08	ND	0.96	1.89
Eicosatrienoic acid; C_20:3_	0.31 ± 0.07 ns	ND	ND	ND	1.79 ± 1.55 ns	ND	0.34	ND	0.24	1.00	0.32	0.36
Behenic acid; C_22:0_	0.23 ± 0.16 ns	0.66 ± 0.58 ns	5.87 ± 5.20 ns	0.29 ± 0.03 ns	ND	0.98 ± 0.92 ns	0.35	1.03	ND	ND	0.15	1.11
Erucic acid; C_22:1_	0.12 ± 0.03 ns	0.16 ± 0.15 ns	ND	ND	ND	0.27 ± 0.15 ns	2.38	0.15	0.06	ND	0.11	0.19
Lignoceric acid; C_24:0_	0.33 ± 0.12 b	1.01 ± 0.13 ab	3.64 ± 0.18 a	0.26 ± 0.12 b	1.18 ± 0.21 ab	1.62 ± 0.32 ab	0.52	1.78	0.10	1.00	0.40	1.21
Nervonic acid; C_24:1_	0.90 ± 0.21 ab	0.51 ± 0.03 b	1.81 ± 0.68 a	ND	ND	ND	0.46	ND	0.20	ND	0.07	0.45
Total NDFA	10.94 ± 0.78	27.03 ± 1.66	18.28 ± 3.17	6.68 ± 4.39	12.18 ± 4.02	7.81 ± 2.68	6.35	4.96	1.04	3.00	2.57	10.38
Total DFA + NDFA	90.18 ± 5.04	92.99 ± 0.25	96.25 ± 0.14	87.83 ± 7.50	95.97 ± 0.81	86.25 ± 5.22	89.45	96.54	98.48	98.00	92.05	91.09

^†^ Values are expressed as percentage of total fatty acids. Means with different letters within the same row are significantly different (*p* < 0.05); ns: not statistically significant. ^‡^ DFA, dominant fatty acids. ^£^ NDFA, non-dominant fatty acids. ^¤^ Results are not included in analysis of variance due to excessive standard deviation. ^¥^ ND, not detected.

## Data Availability

The data that support the findings of this study are available from the corresponding author on request.

## Supplementary Materials

The following are available online at https://www.mdpi.com/article/10.3390/foods10092229/s1, Supplementary Figure S1. Flowers of botanical origins for bee pollen samples. (1) *Brassica napus*, (2) *Bidens pilosa* var. *radiata*, (3) *Camellia sinensis*, (4) *Fraxinus griffithii*, (5) *Prunus mume*, (6) *Rhus chinensis* var. *roxburghii*, (7) *Bombax ceiba*, (8) *Hylocereus costaricensis*, (9) *Liquidambar formosana*, (10) *Nelumbo nucifera*, (11) *Zea mays.* Supplementary Table S1. Chemical score in bee pollens compared to the minimum requirements of honey bees. Supplementary Table S2. Chemical score in bee pollens compared to the minimum requirements of adult humans.
