# Supplementary material for: Nutritive Value of 11 Bee Pollen Samples from Major Floral Sources in Taiwan
Source: Foods. 2021 Sep 20;10(9):2229. doi: 10.3390/foods10092229 (PMC8469103; doi:10.3390/foods10092229)
Supplement: Supplementary file 1 [file foods-10-02229-s001.zip › foods-1349924-supplementary.pdf]

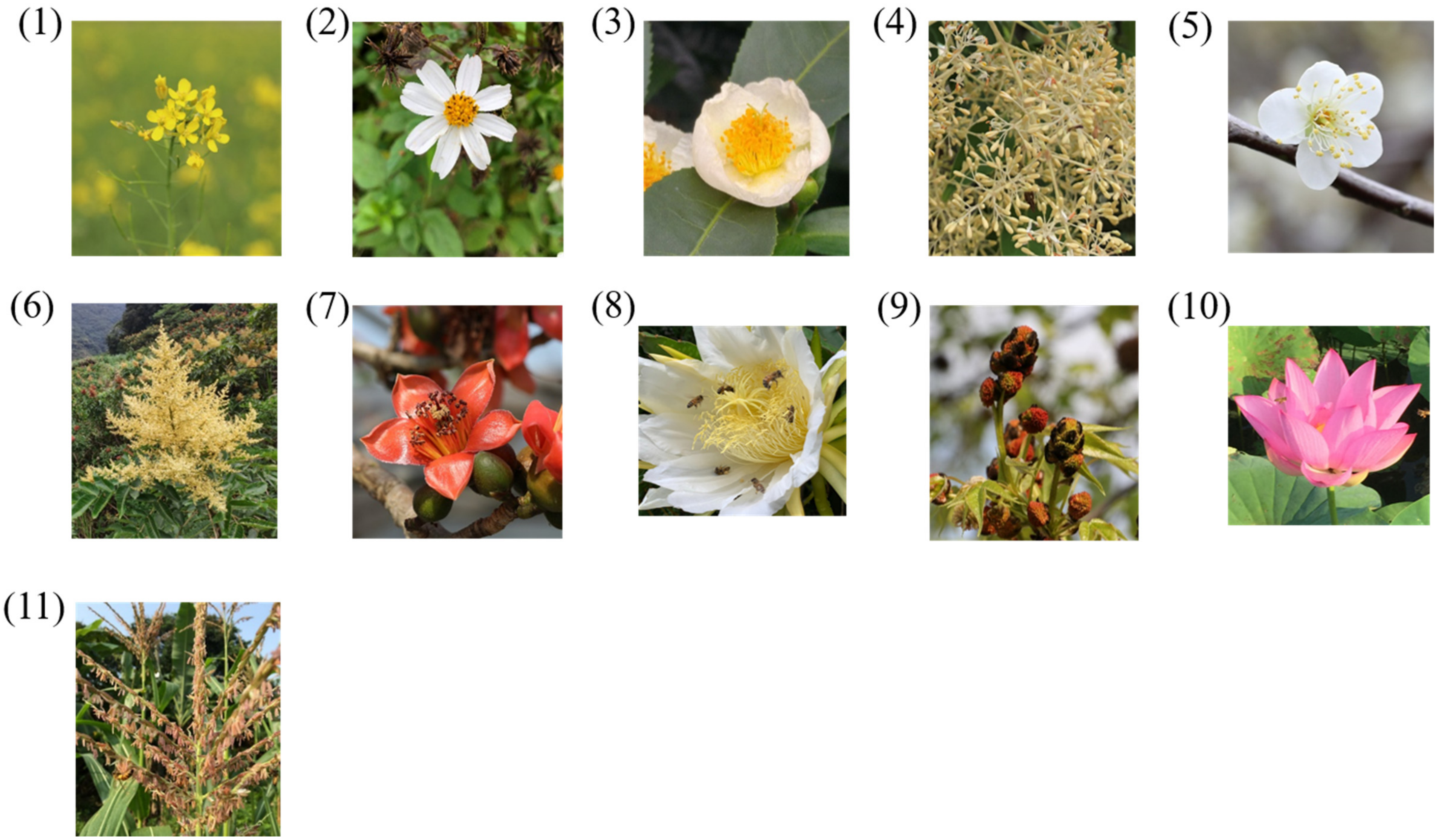

Figure S1. Flowers of botanical origins for bee pollen samples. (1) *Brassica napus*, (2) *Bidens pilosa* var. *radiata*, (3) *Camellia sinensis*, (4) *Fraxinus griffithii*, (5) *Prunus mume*, (6) *Rhus chinensis* var. *roxburghii*, (7) *Bombax ceiba*, (8) *Hylocereus costaricensis*, (9) *Liquidambar formosana*, (10) *Nelumbo nucifera*, (11) *Zea mays.*

Table S1. Chemical score in bee pollens compared to the minimum requirements of honey bees.

Amino acids	Bn	Bp	Cs	Fg	Pm	Rc	Bc	Hc	Lf	Nn	Zm	Average
Arginine	158.81	116.43	151.64	145.85	145.28	129.68	150.10	167.83	213.15	170.16	143.78	153.88
Histidine	114.39	297.73	164.17	213.83	136.07	133.56	166.23	146.15	65.26	182.47	117.95	157.98
Isoleucine	111.22	104.18	113.25	114.11	101.19	103.69	116.80	134.48	109.39	130.47	97.84	112.42
Leucine	148.38	143.33	153.78	151.13	138.28	139.07	141.57	168.95	173.27	162.18	133.13	150.28
Lysine	233.68	202.43	196.79	192.80	188.44	204.62	187.78	245.94	261.70	232.61	194.08	212.81
Methionine	86.51	83.20	89.75	92.16	64.78	85.59	99.43	109.19	110.57	102.08	45.68	88.09
Phenylalanine	271.66	325.82	292.20	284.10	265.91	279.10	267.54	331.23	242.68	288.27	224.91	279.40
Threonine	259.19	231.80	239.04	241.65	231.65	237.73	246.37	267.56	214.01	264.98	245.34	243.58
Tryptophan	101.18	107.92	100.64	62.66	77.12	186.97	61.99	125.24	58.84	75.18	55.90	92.15
Valine	120.80	128.26	130.78	132.72	127.40	124.25	139.36	166.16	46.33	146.62	144.60	127.93
Average	160.58	174.11	163.20	163.10	147.61	162.43	157.72	186.27	149.52	175.50	140.32	161.85

Table S2. Chemical score in bee pollens compared to the minimum requirements of adult humans.

Amino acids	Bn	Bp	Cs	Fg	Pm	Rc	Bc	Hc	Lf	Nn	Zm	Average
Histidine	114.39	297.73	164.17	213.83	136.07	133.56	166.23	146.15	65.26	182.47	117.95	157.98
Isoleucine	148.30	138.91	151.00	152.15	134.92	138.26	155.74	179.31	145.85	173.96	130.45	149.89
Leucine	113.17	109.32	117.29	115.27	105.47	106.07	107.97	128.86	132.16	123.70	101.54	114.62
Lysine	155.79	134.95	131.19	128.54	125.62	136.41	125.19	163.96	174.47	155.07	129.39	141.87
Methionine	81.10	78.00	84.14	86.40	60.74	80.24	93.21	102.37	103.66	95.70	42.83	82.58
Threonine	169.04	151.17	155.90	157.60	151.08	155.04	160.68	174.49	139.57	172.81	160.01	158.85
Tryptophan	168.63	179.87	167.73	104.43	128.54	311.62	103.32	208.74	98.07	125.29	93.16	153.58
Valine	123.90	131.55	134.13	136.13	130.67	127.43	142.93	170.42	47.51	150.38	148.31	131.21
Phenylalanine + Tyrosine	162.58	184.49	172.91	175.78	164.44	172.94	165.42	191.90	125.26	193.99	145.87	168.69
Average	137.43	156.22	142.05	141.12	126.39	151.29	135.63	162.91	114.65	152.60	118.83	139.92
